# Supplementary material for: Proteome Mapping of South African Cassava Mosaic Virus-Infected Susceptible and Tolerant Landraces of Cassava
Source: Proteomes. 2021 Oct 23;9(4):41. doi: 10.3390/proteomes9040041 (PMC8628908; doi:10.3390/proteomes9040041)
Supplement: Supplementary file 1 [file proteomes-09-00041-s001.zip › Legend Figure S1.pdf]

Legend Figure S1

Figure S1. TME3 differential expressed protein interactions at 67 dpi. (**a**) STRING protein-protein interaction network functional enrichment analysis of over-expressed proteins with *p*-value < 1.0e-16. Network Settings: Full network; Confidence edge; Text mining; Experiments; Databases; Co-expression Interaction sources; Confidence of 0.7; More than 50 interactors 1st and 2nd shell; Hide disconnected nodes in the network (**b**) KEGG pathways analysis of over-expressed and under-expressed proteins with false discovery rate (FDR) < 0.05.
