# Supplementary material for: Proteome Mapping of South African Cassava Mosaic Virus-Infected Susceptible and Tolerant Landraces of Cassava
Source: Proteomes. 2021 Oct 23;9(4):41. doi: 10.3390/proteomes9040041 (PMC8628908; doi:10.3390/proteomes9040041)
Supplement: Supplementary file 1 [file proteomes-09-00041-s001.zip › Supplementary Files/Supplementary Tables_Proteomes_1310851_Ramulifho and Rey 2021 (2).pdf]

Proteome mapping of tolerant TME3 and susceptible T200 cassava landraces infected with South African cassava mosaic virus

Elelwani Ramulifho [1,2], Chrissie Rey [1,*]

Supplementary Materials

Table S1. Primers used for RT-qPCR

Gene Name	Primer Sequences
UBQ10	**Fwd**: 5′ TGCATCTCGTTCTCCGATTG 3′
	**Rev**: 5′ GCGAAGATCAGTCGTTGTTGG 3′
Manes.05G194900 (Receptor like kinase_1)	**Fwd**: 5′ AGTCAGACCTCATCGACCCA 3′
	**Rev**: 5′ AACCTGAGCAACTCGACCAG 3′
Manes.06G055700 (Receptor like protein)	**Fwd**: 5′ GCCCCTACCTTCACAGCATT 3′
	**Rev**: 5′ AACAGCAGCAGTCTCAGCAT 3′
Manes.08G126700 (Kinase)	**Fwd**: 5′ GTGGGCCTGTAAAGGATGCT 3′
	**Rev**: 5′ TCGATGCCGAGACAAACCAA 3′
Manes.14G124000 (Receptor like kinase_2)	**Fwd**: 5′ TCCACCATTAACCTGGGCAC 3′
	**Rev**: 5′ CCCATGAGGAACAGCACGAT 3′
Manes.18G115700 (LRR)	**Fwd**: 5′ GCCTCAAACTCACCTCAGCT 3′
	**Rev**: 5′ GCTGAGCATAGCAGATGGGT 3′
Manes.08G075100 (C3HC4-type RING finger)	**Fwd**: 5′ CCGTTTTATGCCCCTCCTGT 3′
	**Rev**: 5′ TCTCACCTCTGGCCTCTCTC 3′
Manes.15G008400 (Pathogenesis-related protein_1)	**Fwd**: 5′ TGAAGGTGATCCGTGGTTGG 3′
	**Rev**: 5′ TGGTGCACTTGCTGATGGAT 3′
Manes.18G143800 (*LRX2*)	**Fwd**: 5′ ATTTCCGGGATCACCACCAC 3′
	**Rev**: 5′ CTGTTGCTGAGGAGGTGGAG 3′
Manes.01G100900 (*SnRK1*.1)	**Fwd**: 5′ AGAAGTCCTCAAAGCCCTGC 3′
	**Rev**: 5′CCTGGAATCCACCTGCACTT 3′
Manes.15G007900 (Pathogenesis-related protein_2)	**Fwd**: 5′ TCCGGCAAAGCTGTACAGAG 3′
	**Rev**: 5′ TTCTTGATGGTTCCAGGCCC 3′

Table S2. Gene Ontology annotation of differentially expressed proteins of T200 and TME3 at 32 dpi.

GO functional groups	T200 OE[a]	T200 UE[b]	TME3 OE	TME3 UE
**Cellular Component**				
Cell wall	16%	13%	14%	15%
Chloroplast	16%	50%	27%	44%
Cytoplasm	58%	40%	36%	56%
Cytosol	16%	40%	32%	30%
Endoplasmic reticulum	-	3%	-	4%
Endosome	-	3%	5%	-
External encapsulating structure	-	3%	-	4%
Extracellular region	21%	23%	14%	30%
Golgi apparatus	-	3%	-	4%
Mitochondrion	37%	13%	23%	22%
Nucleus	11%	33%	18%	30%
Other cellular components	11%	10%	5%	15%
other intracellular components	32%	23%	32%	22%
other membranes	21%	17%	18%	19%
Peroxisome	-	3%	-	4%
Plasma membrane	16%	27%	23%	22%
Plastid	26%	40%	27%	41%
Thylakoid	-	3%	5%	-
Vacuole	-	13%	5%	15%
Unknown cellular components	-	3%	5%	-
**Biological Process**				
Anatomical structure development	-	11%	-	13%
Biosynthetic process	20%	26%	5%	39%
Carbohydrate metabolic process	7%	11%	-	17%
Catabolic process	20%	22%	26%	17%
Cell communication	7%	-	5%	-
Cell cycle	-	4%	5%	-
Cellular component organization	7%	15%	11%	13%
Cellular protein modification process	7%	4%	-	9%
Circadian rhythm	-	4%	-	4%
DNA metabolic process	-	4%	5%	-
Embryo development	-	4%	-	4%
Flower development	-	4%	-	4%
Generation of precursor metabolites and energy	7%	7%	5%	9%
Lipid metabolic process	7%	15%	5%	17%
Multicellular organism development	7%	19%	5%	22%
Nucleobase-containing compound metabolic process	-	15%	-	13%
Other biological processes	-	4%	-	4%
Other cellular processes	67%	59%	53%	70%
Other metabolic processes	60%	55%	47%	65%

Post-embryogenic development	-	7%	-	9%
Protein metabolic process	20%	11%	11%	17%
Regulation of molecular function	-	4%	-	4%
Reproduction	-	15%	5%	13%
Response to abiotic stimulus	-	26%	11%	22%
Response to biotic stimulus	13%	7%	5%	13%
Response to chemical	20%	7%	5%	17%
Response to endogenous stimulus	7%	-	-	4%
Response to external stimulus	20%	11%	11%	17%
Response to light stimulus	7%	11%	-	17%
Response to stress	33%	30%	37%	26%
Secondary metabolic process	13%	4%	5%	9%
Signal transduction	7%	8%	5%	9%
Translation	-	8%	-	9%
Transport	-	8%	11%	-
Unknown biological processes	-	8%	5%	4%
**Molecular Function**				
Catalytic activity	57%	32%	47%	38%
DNA binding	-	4%	-	4%
Enzyme regulator activity	-	4%	-	4%
Hydrolase activity	36%	24%	33%	25%
Kinase activity	-	4%	-	4%
Lipid binding	-	4%	-	4%
Nucleic acid binding	-	4%	-	4%
Nucleotide binding	21%	8%	13%	13%
Other binding	43%	36%	40%	38%
Protein binding	7%	24%	7%	25%
RNA binding	-	16%	7%	13%
Transferase activity	29%	16%	13%	25%
Transporter activity	7%	4%	-	8%
Unknown molecular functions	-	8%	13%	4%

[a] OE is short for over-expressed differentially expressed proteins

[b] UE is short for under-expressed differentially expressed proteins

Table S3. Gene Ontology annotation of differentially expressed proteins of T200 and TME3 at 67 dpi.

GO functional groups	T200 OE[a]	T200 UE[b]	TME3 OE	TME3 UE
**Cellular Component**				
Cell wall	9%	21%	13%	12%
Chloroplast	45%	48%	56%	31%
Cytoplasm	47%	34%	44%	43%
Cytoskeleton	1%	-	-	1%
Cytosol	50%	38%	53%	40%
Endoplasmic reticulum	9%	9%	6%	14%
Endosome	1%	-	-	2%
External encapsulating structure	1%	2%	1%	1%
Extracellular region	12%	33%	17%	20%
Golgi apparatus	10%	9%	8%	12%
Mitochondrion	20%	24%	24%	17%
Nuclear envelope	1%	-	-	2%
Nucleolus	2%	5%	3%	4%
Nucleus	29%	31%	26%	35%
Other cellular components	17%	14%	12%	22%
Other intracellular components	23%	29%	25%	25%
Other membranes	18%	22%	18%	21%
Peroxisome	4%	5%	5%	2%
Plasma membrane	20%	28%	22%	22%
Plastid	25%	29%	32%	17%
Ribosome	2%	14%	6%	5%
Thylakoid	6%	16%	13%	4%
Vacuole	16%	12%	16%	12%
Unknown cellular components	-	-	-	1%
**Biological Process**				
Anatomical structure development	17%	16%	12%	24%
Biosynthetic process	36%	22%	35%	28%
Carbohydrate metabolic process	10%	8%	13%	4%
Catabolic process	17%	12%	19%	12%
Cell communication	6%	6%	5%	9%
Cell cycle	2%	-	-	3%
Cell death	3%	2%	1%	6%
Cell differentiation	4%	4%	5%	3%
Cell growth	4%	-	3%	3%
Cellular component organization	11%	16%	11%	15%
Cellular homeostasis	1%	-	-	1%
Cellular protein modification process	9%	24%	14%	12%
Circadian rhythm	2%	4%	3%	1%
DNA metabolic process	1%	2%	1%	1%
Embryo development	2%	6%	2%	4%

Generation of precursor metabolites and energy	1%	8%	8%	-
Flower development	4%	2%	-	3%
Growth	5%		4%	3%
Lipid metabolic process	11%	2%	6%	12%
Multicellular organism development	17%	16%	13%	22%
Nucleobase-containing compound metabolic process	12%	20%	13%	18%
Other biological processes	5%	-	3%	4%
Other cellular processes	77%	62%	71%	75%
Other metabolic processes	63%	54%	67%	51%
Photosynthesis	2%	8%	6%	-
Pollination	2%	2%	3%	1%
Post-embryogenic development	8%	6%	5%	12%
Protein metabolic process	9%	20%	13%	10%
Regulation of gene expression, epigenetic	-	2%	1%	-
Regulation of molecular function	-	2%	-	1%
Reproduction	9%	8%	6%	12%
Response to abiotic stimulus	22%	16%	22%	19%
Response to biotic stimulus	8%	6%	9%	12%
Response to chemical	35%	20%	32%	28%
Response to endogenous stimulus	13%	10%	11%	13%
Response to external stimulus	12%	8%	13%	13%
Response to light stimulus	6%	7%	5%	9%
Response to stress	31%	24%	31%	28%
Secondary metabolic process	4%	-	3%	3%
Signal transduction	7%	8%	5%	10%
Translation	5%	12%	6%	9%
Transport	14%	14%	11%	19%
Tropism	1%	-	-	1%
Unknown biological processes	4%	8%	6%	4%
**Molecular Function**				
Carbohydrate binding	2%	4%	4%	-
Catalytic activity	38%	41%	46%	28%
Chromatin binding	1%	2%	1%	-
DNA binding	3%	6%	3%	1%
Enzyme regulator activity	22%	4%	3%	3%
Hydrolase activity	6%	22%	27%	15%
Kinase activity	3%	6%	8%	4%
Lipid binding	1%	6%	3%	4%
Nuclease activity	9%	-	-	1%
Nucleotide binding	36%	6%	8%	7%
Other binding	2%	20%	31%	32%
Other molecular functions	1%	-	1%	3%
Protein binding	23%	27%	23%	25%

RNA binding	12%	24%	13%	20%
Signalling receptor activity	1%	2%	1%	-
Structural molecule activity	3%	8%	3%	7%
Transcription regulator activity	1%	-	-	1%
Transferase activity	24%	14%	25%	15%
Translation factor activity, RNA binding	2%	-	-	3%
Transporter activity	5%	-	2%	6%
Unknown molecular functions	5%	6%	5%	-

[a] OE is short for over-expressed differentially expressed proteins

[b] UE is short for under-expressed differentially expressed proteins
