# Supplementary figures and images for: Proteome Mapping of South African Cassava Mosaic Virus-Infected Susceptible and Tolerant Landraces of Cassava
Source: Proteomes. 2021 Oct 23;9(4):41. doi: 10.3390/proteomes9040041 (PMC8628908; doi:10.3390/proteomes9040041)

(a)

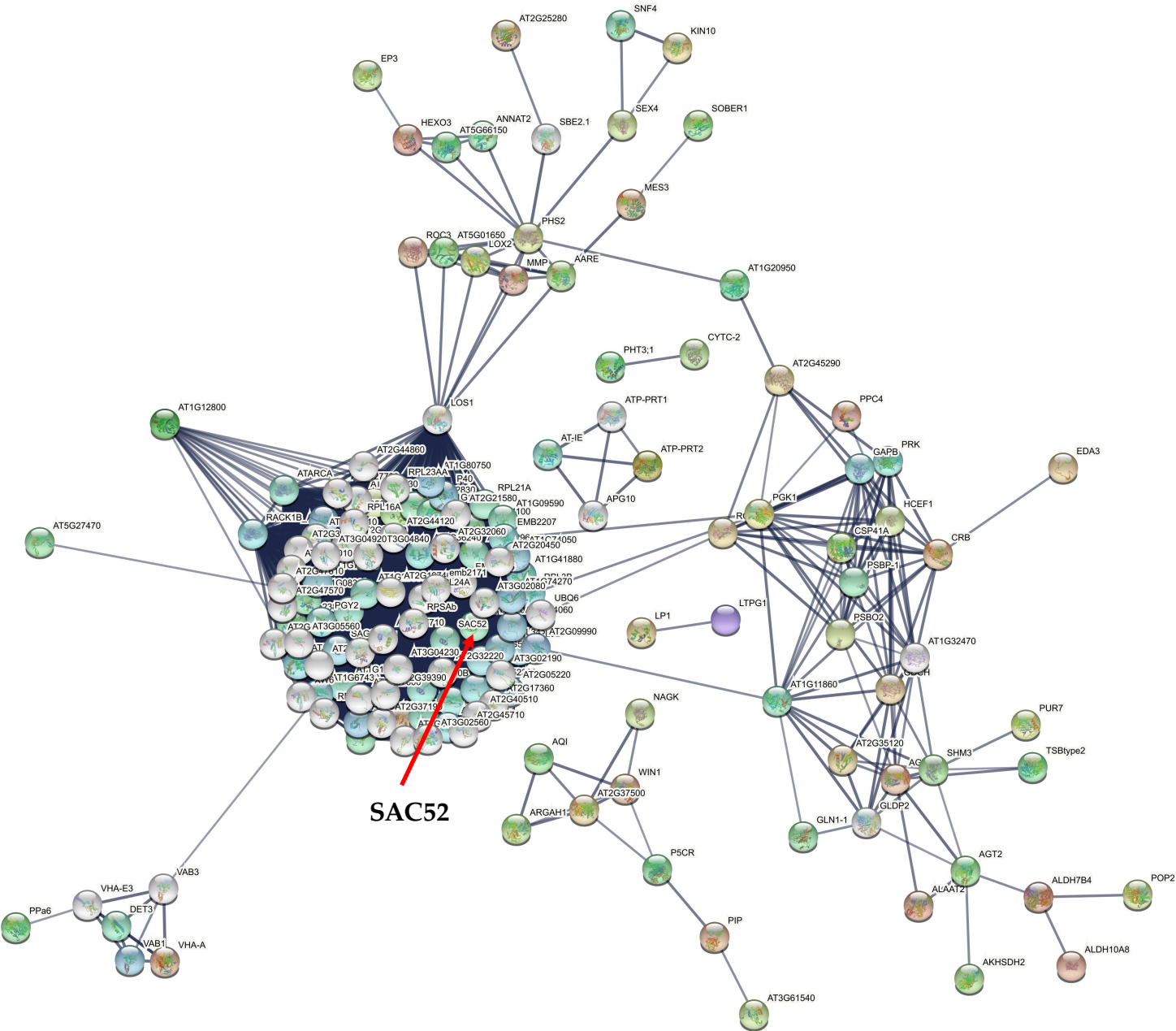

(b)

KEGG: TME3 67 dpi

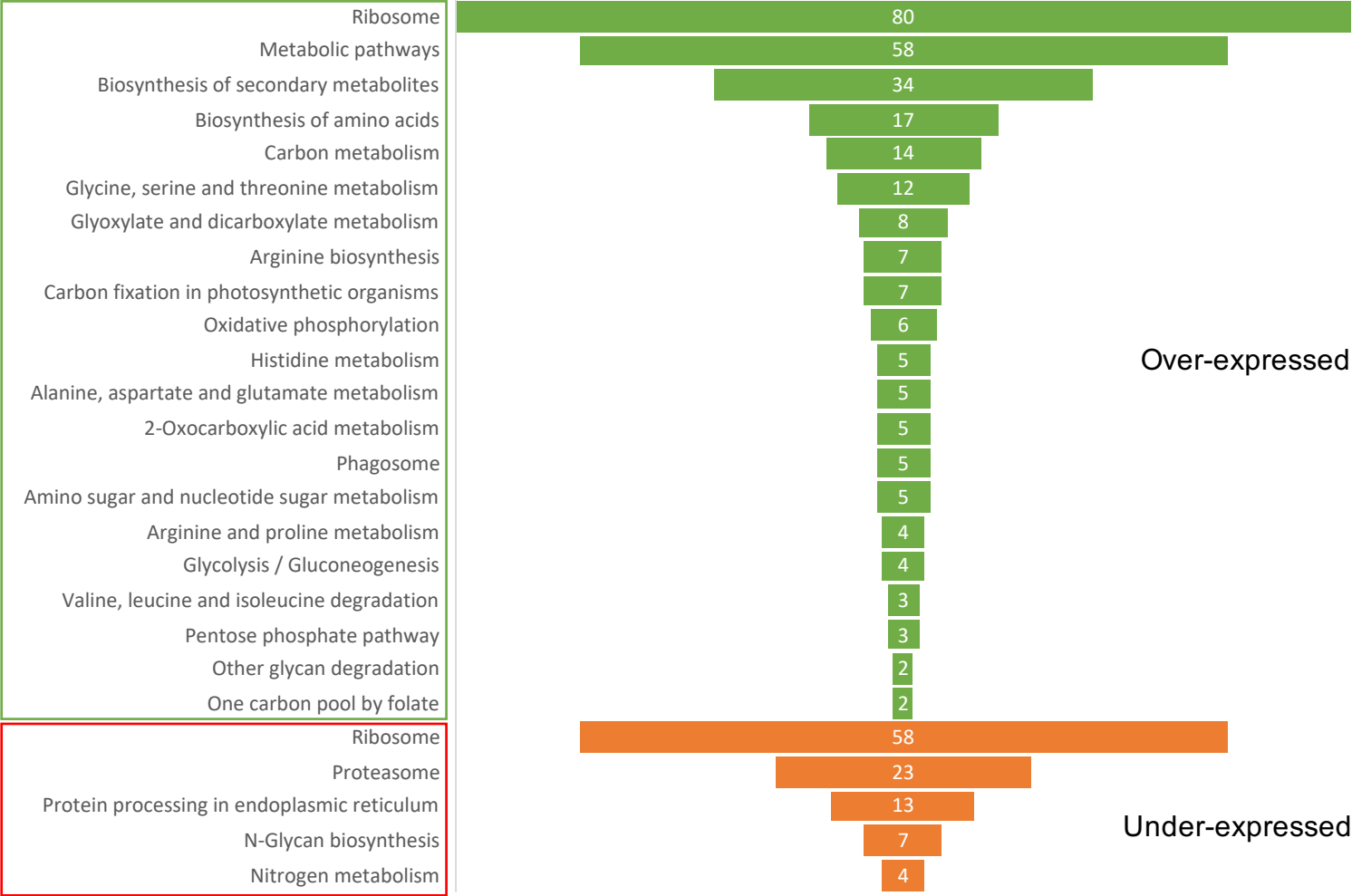

Supplement: Supplementary file 1 [file proteomes-09-00041-s001.zip › Supplementary Files/Figure S1 revised.pdf]
